# Development of Age and Gender Estimation Standards From Sternal Anthropometry: An Autopsy-Based Correlation Study in the Central Indian Population (Indore Region)

**DOI:** 10.7759/cureus.87884

**Published:** 2025-07-14

**Authors:** Pankaj Nema, Anil Mangeshkar, Shubhanshu Gupta, Priyanka K Nema, Sameer Sathe, Sanjay Dadu

**Affiliations:** 1 Department of Forensic Medicine and Toxicology, Sri Aurobindo Medical College and Postgraduate Institute, Indore, IND; 2 Department of Forensic Medicine and Toxicology, Government Medical College, Datia, IND; 3 Department of Community Medicine, Government Medical College, Datia, IND; 4 Department of Ophthalmology, Employees' State Insurance Corporation Medical College, Indore, IND; 5 Department of Anatomy, Government Medical College, Datia, IND; 6 Department of Forensic Medicine, Nandkumar Singh Chouhan Government Medical College, Khandwa, IND

**Keywords:** age estimation, central indian population, forensic anthropology, forensic identification, skeletal fusion, sternum

## Abstract

Objective: This study aims to develop region-specific, autopsy-based anthropometric standards of the sternum for age and gender estimation in the Central Indian population. The goal is to improve the accuracy of forensic identification, particularly in cases involving incomplete or decomposed remains.

Methods: A total of 770 sternum (432 males, 338 females) were analyzed for age estimation; 632 were also assessed for sex determination. Measurements included manubrium length, mesosternum length, combined sternal length, widths of the first and third sternebrae, sternal index, and relative width index. The fusion of the manubrium to the mesosternum and the xiphoid process to the body was documented. Data were analyzed using Statistical Product and Service Solutions (SPSS, version 23.0; IBM SPSS Statistics for Windows, Armonk, NY), employing descriptive statistics, Pearson’s correlation, and linear regression.

Results: Fusion of the second to the first sternum showed a statistically significant correlation with age (p < 0.0001), with fusion generally complete by 35 years. Fusion of the manubrium to the body exhibited variable patterns. Fusion of the xiphoid to the body began at a mean age of 43.70 years in males and 49.67 years in females, with incomplete fusion observed beyond 60 years. Comparison with existing studies revealed regional variation in sternal fusion patterns.

Conclusion: The sternum demonstrates significant age-related morphological changes with identifiable fusion patterns useful for forensic age estimation. However, variability in the fusion timing suggests that sternal analysis should be used with other skeletal indicators. These findings establish regional anthropometric standards for forensic applications in Central India.

## Introduction

Traditionally, the cranial and pelvic bones, followed by long bones, are considered the most reliable skeletal elements for estimating age and sex in human remains [1]. However, in many forensic cases involving decomposed, mutilated, or incomplete remains, these key skeletal structures may not be retrievable. Forensic anthropologists and medico-legal experts often rely on less sexually dimorphic bones, such as the sternum, for crucial information [2].

The concept of personal identity is legally recognized under Article 6 of the Universal Declaration of Human Rights, which affirms the right of every individual to recognition before the law [3]. Establishing identity and confirming the corpus delicti - particularly in cases involving assault, homicide, rape, disputed paternity, or civil matters such as inheritance and marriage - remains fundamental to both criminal and civil justice systems worldwide [4].

Among skeletal elements, the sternum - due to its compact structure and resistance to putrefaction - frequently endures in forensic contexts where other bones may deteriorate [5]. Specifically, differences in the ratio of manubrium to mesosternum lengths, first described by Hyrtl, offer a practical approach for distinguishing between male and female sterna [6].

Studies have demonstrated that specific sternal dimensions - especially the mesosternum and combined sternal length - significantly differentiate sexes [7]. Moreover, discriminant function analysis and limiting point techniques have been used to enhance the accuracy of these estimations [8]. Determining the age of human skeletal remains also remains a core objective in forensic and medico-legal investigations. While near-perfect accuracy (~90%) can be achieved using cranial and pelvic bones, estimation from the sternum alone remains challenging and requires population-specific standards [9]. Age-related sternal changes - including patterns of ossification and fusion of sternebrae - can be quantified and correlated with chronological age to improve accuracy [10].

Given the known variability in skeletal dimensions across different ethnic and geographical groups, it is essential to establish local standards for forensic application [11]. Applying data derived from other populations to the Central Indian population may result in significant inaccuracies in age estimation, which can adversely affect medico-legal conclusions. The sternum’s durability, even in advanced decomposition, mutilation, or fragmentation stages, makes it an indispensable element in forensic cases involving incomplete remains. Thus, developing validated anthropometric standards and formulae for this population will enhance the reliability of forensic identification processes.

Therefore, the present study aims to develop population-specific anthropometric standards of the sternum for accurate age and sex estimation in the Central Indian population using autopsy-derived samples. By analyzing sternal dimensions and fusion patterns across age groups, the study seeks to establish reliable forensic reference data applicable in cases involving decomposed, mutilated, or incomplete remains.

## Materials and methods

Study design and setting

This analytical study was conducted over 12 months, from July 2016 to June 2017, in the Department of Forensic Medicine and Toxicology mortuary at M.G.M. Medical College, Indore, Madhya Pradesh, India. The primary aim was to assess the sternum's anthropometric characteristics for forensic age and sex estimation in the Central Indian population.

Sample size and criteria

A total of 770 sterna were included for age estimation, comprising 432 males and 338 females. Of these, 632 sterna (350 male, 282 female) were analyzed for sex determination. All sterna that met the inclusion criteria over the 12 months were included to enhance the statistical reliability of the analyses. Additionally, the minimum required sample size was estimated using the formula:

\[ n = \frac{Z^2 \cdot p \cdot (1 - p)}{d^2} \]

Here, n is the required sample size, Z is the Z-value (1.96 for 95% confidence), p is the expected proportion of the population with the characteristic of interest (assumed to be 0.5 to maximize sample size), and d is the margin of error (typically 0.05). Based on this formula, the minimum required sample size was approximately 384. Still, to increase precision and allow subgroup analyses by age and sex, the final sample was expanded to include all eligible cases, as done in similar osteological studies [12].

The cases in which the age of the deceased was known and was above 10 years were included. Sterna showing pathological lesions, healed or recent fractures, gross deformities, or missing parts were excluded to minimize measurement bias.

Materials and measurements

All sterna were collected during medico-legal autopsies and carefully dissected by removing the overlying soft tissues, peristernal fascia, and attached costal cartilages. After dissection, the bones were thoroughly rinsed with water to eliminate blood and tissue remnants. They were then preserved in a moist state by wrapping them in saline-soaked gauze until the time of measurement, ensuring the bone's anatomical integrity and natural dimensions. These moist, anatomically fresh specimens are called “wet sterna,” distinguishing them from dried or macerated bones typically used in osteoarchaeological studies.

Anthropometric measurements were obtained using standard tools, including a precision sliding Vernier caliper accurate to the nearest millimeter, a flexible measuring tape, and a measuring scale. All observations were recorded along the mid-sagittal plane of the sternum. Manubrium length was measured as the distance from the midpoint of the suprasternal (jugular) notch to the manubrio-mesosternal junction, while mesosternum length extended from the manubrio-mesosternal junction to the xipho-sternal junction. The combined sternal length was calculated by summing the manubrium and mesosternum lengths. The width of the upper part of the body, corresponding to the first sternebra, was measured transversely at the level of articulation with the second costal cartilages. Similarly, the width of the third sternebra, located in the midportion of the mesosternum, was measured between the lateral margins at the level of articulation with the fourth and fifth costal cartilages. These width measurements reflect the transverse breadth of specific sternebral segments rather than the entire body of the sternum. Additionally, two indices were derived: the sternal index, calculated by dividing manubrium length by mesosternum length and multiplying by 100, and the relative width index, calculated by dividing the width of the first sternebra by that of the third sternebra and multiplying by 100.

Before data collection, the examiner underwent standardized training in anthropometric techniques under the supervision of an experienced forensic anthropologist to ensure consistency in landmark identification and measurement procedures. Instruments were calibrated daily using a precision metal gauge to maintain measurement accuracy. To assess intra-observer reliability, a subset of 30 sterna was re-measured after a two-week interval. The intraclass correlation coefficient (ICC) for the repeated measurements exceeded 0.95 for all parameters, indicating excellent measurement reliability.

Age verification and fusion assessment

The age of each deceased individual was verified through official police records, family-provided statements, and identification documents. For analytical consistency, the recorded age was rounded to the nearest whole year. In addition to the linear measurements, each sternum was macroscopically examined for the fusion status at two anatomical junctions: the manubrio-mesosternal and xipho-sternal joints. Fusion status at each site was categorized as complete, partial, or absent. Complete fusion was a continuous bony bridge across the joint line with no visible gap or separation. Partial fusion was identified when there was evidence of bony bridging or coalescence at one or more points along the articulation, but with intervening gaps or incomplete continuity. Absent fusion referred to a clear, unfused joint line with no visible ossification between adjoining segments. Under adequate lighting conditions, these criteria were applied uniformly across all specimens during visual and tactile inspection.

Statistical analysis

All data were compiled and organized using Microsoft Excel (Microsoft Corp., Redmond, WA) and analyzed using Statistical Product and Service Solutions (SPSS, version 23.0; IBM SPSS Statistics for Windows, Armonk, NY). Descriptive statistics, including mean, standard deviation, range, and standard error of the mean, were calculated for continuous variables. Categorical variables such as fusion status were expressed as frequencies and percentages. The normality of continuous data was assessed using the Shapiro-Wilk test and visual inspection of histograms and Q-Q plots. Pearson’s correlation was used to assess associations between sternal measurements and age. An independent (unpaired) t-test was applied to compare mean sternal measurements between male and female groups. Linear regression analysis was performed to derive predictive equations for age estimation using individual sternal dimensions and calculated indices, including the sternal index and relative width index. A p-value less than 0.05 was considered statistically significant for all analyses.

## Results

The analysis of fusion between the fourth and third sternal segments across different age groups revealed that complete fusion was the most commonly observed pattern. The highest frequency of complete fusion was noted in the 31-35 years age group, accounting for 99 cases (12.86%), followed by 92 cases (11.98%) in the 26-30 years group and 82 cases (10.65%) in the 21-25 years group. Non-fusion was extremely rare, observed in only two cases (100.0%), both of which belonged to the 26-30 years age group. Partial fusion was not observed in any age category. Statistical analysis showed no significant association between age and fusion status (p > 0.05), suggesting that the distribution of fusion between these sternal segments did not differ significantly across age groups (Table 1).

**Table 1:** Distribution of fusion between fourth and third sternal segments across age groups

Age group (years)	Complete fusion n (%)	Partial fusion n (%)	Non-fusion n (%)	Total n (%)
< 15	14 (1.82%)	0 (0.0%)	0 (0.0%)	14 (1.82%)
16–20	82 (10.68%)	0 (0.0%)	0 (0.0%)	82 (10.65%)
21–25	82 (10.68%)	0 (0.0%)	0 (0.0%)	82 (10.65%)
26–30	92 (11.98%)	0 (0.0%)	2 (100.0%)	94 (12.20%)
31–35	99 (12.89%)	0 (0.0%)	0 (0.0%)	99 (12.86%)
36–40	76 (9.90%)	0 (0.0%)	0 (0.0%)	76 (9.87%)
41–45	79 (10.29%)	0 (0.0%)	0 (0.0%)	79 (10.26%)
46–50	61 (7.94%)	0 (0.0%)	0 (0.0%)	61 (7.92%)
51–55	65 (8.46%)	0 (0.0%)	0 (0.0%)	65 (8.44%)
56–60	66 (8.59%)	0 (0.0%)	0 (0.0%)	66 (8.57%)
> 60	52 (6.77%)	0 (0.0%)	0 (0.0%)	52 (6.75%)
Total	768 (100.0%)	0 (0.0%)	2 (100.0%)	770 (100.0%)

The fusion distribution between the second and first sternal segments across age groups revealed that complete fusion was predominant in most categories. The highest frequencies were observed in the 31-35 years group with 97 (13.43%) cases, followed by 94 (13.02%) cases in the 26-30 years group and 76 (10.53%) cases in the 21-25 years group. Non-fusion was most frequently noted in the <15 years group with 14 (31.82%) cases and in the 16-20 years group with 24 (54.55%) cases. Partial fusion was rare, reported in only four (100%) cases, primarily within the 16-20 and 21-25 age groups. A statistically significant association was found between age and the degree of fusion (p < 0.05), indicating that fusion patterns between the second and first sternal segments are age-dependent (Table 2).

**Table 2:** Distribution of fusion between second and first sternal segments across age groups

Age group (years)	Complete fusion N (%)	Partial fusion N (%)	Non-fusion N (%)	Total N (%)
< 15	0 (0.0%)	0 (0.0%)	14 (31.82%)	14 (1.82%)
16–20	56 (7.76%)	2 (50.0%)	24 (54.55%)	82 (10.65%)
21–25	76 (10.53%)	2 (50.0%)	4 (9.09%)	82 (10.65%)
26–30	94 (13.02%)	0 (0.0%)	0 (0.0%)	94 (12.20%)
31–35	97 (13.43%)	2 (4.55%)	0 (0.0%)	99 (12.86%)
36–40	76 (10.53%)	0 (0.0%)	0 (0.0%)	76 (9.87%)
41–45	79 (10.94%)	0 (0.0%)	0 (0.0%)	79 (10.26%)
46–50	61 (8.45%)	0 (0.0%)	0 (0.0%)	61 (7.92%)
51–55	65 (9.00%)	0 (0.0%)	0 (0.0%)	65 (8.44%)
56–60	66 (9.14%)	0 (0.0%)	0 (0.0%)	66 (8.57%)
> 60	52 (7.20%)	0 (0.0%)	0 (0.0%)	52 (6.75%)
Total	722 (100.0%)	4 (100.0%)	44 (100.0%)	770 (100.0%)

The distribution of fusion between the manubrium and the sternal body across age groups revealed that complete fusion was most frequently observed in the 51-55 years group, with 41 (23.6%) cases, followed by the >60 years group with 36 (20.69%) cases and the 36-40 years group with 22 (12.64%) cases. Partial fusion was most common in the 41-45 years group with 15 (40.54%) cases, followed by seven (18.92%) cases in the 21-25 years group and five (13.51%) cases in the 46-50 years group. Non-fusion was predominantly seen in younger age groups, particularly in the 16-20 years group with 76 (13.60%) cases, the 21-25 years group with 75 (13.42%) cases, and the 26-30 years group with 78 (13.95%) cases. A statistically significant association was found between age and the degree of fusion (p < 0.05), indicating that fusion between the manubrium and sternal body progresses with age (Table 3).

**Table 3:** Relationship between age and the fusion of the manubrium with the sternal body

Age	Fusion of manubrium to body			
	Complete N (%)	Partial N (%)	Non-fusion N (%)	Total N (%)
10-15 year	0 (0.0%)	0 (0.0%)	14 (2.50%)	14 (1.82%)
16-20 year	6 (3.45%)	0 (0.0%)	76 (13.60%)	82 (10.65%)
21-25 year	0 (0.0%)	7 (18.92%)	75 (13.42%)	82 (10.65%)
26-30 year	14 (8.05%)	2 (5.41%)	78 (13.95%)	94 (12.2%)
31-35 year	20 (11.49%)	2 (5.41%)	77 (13.77%)	99 (12.86%)
36-40 year	22 (12.64%)	0 (0.0%)	54 (9.66%)	76 (9.87%)
41-45 year	7 (4.02%)	15 (40.54%)	57 (10.20%)	79 (10.26%)
46-50 year	17 (9.77%)	5 (13.51%)	39 (6.98%)	61 (7.92%)
51-55 year	41 (23.56%)	4 (10.81%)	20 (3.58%)	65 (8.44%)
56-60 year	11 (6.32%)	0 (0.0%)	55 (9.84%)	66 (8.57%)
>60 year	36 (20.69%)	2 (5.41%)	14 (2.50%)	52 (6.75%)
Total	174 (100.0%)	37 (100.0%)	559 (100.0%)	770 (100.0%)

The distribution of fusion between the xiphoid process and the sternal body across age groups showed that complete fusion was most commonly observed in the 51-55 years group with 20 (23.53%) cases, followed by the 41-45, 46-50, and >60 years groups, each with 16 (18.82%) cases. Partial fusion was most frequent in the 51-55 years group with 27 (19.71%) cases, followed by 23 (16.79%) cases in the 56-60 years group and 16 (11.68%) cases in the 31-35 years group. Non-fusion was predominantly seen in younger individuals, especially in the 10-15 years group with 14 (31.82%) cases, the 16-20 years group with 82 (14.96%) cases, and the 21-25 years group with 77 (14.05%) cases. A statistically significant association was observed between age and the degree of fusion (p < 0.05), indicating that fusion of the xiphoid process with the sternal body increases with age (Table 4).

**Table 4:** Relationship between age and fusion of the xiphoid process with the sternal body

Age	Fusion of xiphoid process to body			
	Complete N (%)	Partial N (%)	Non-fusion N (%)	Total N (%)
10-15 years	0 (0.0%)	0 (0.0%)	14 (31.82%)	14 (1.82%)
16-20 years	0 (0.0%)	0 (0.0%)	82 (14.96%)	82 (10.65%)
21-25 years	0 (0.0%)	5 (3.65%)	77 (14.05%)	82 (10.65%)
26-30 years	2 (2.35%)	14 (10.22%)	78 (14.23%)	94 (12.2%)
31-35 years	2 (2.35%)	16 (11.68%)	81 (14.78%)	99 (12.86%)
36-40 years	6 (7.06%)	14 (10.22%)	56 (10.22%)	76 (9.87%)
41-45 years	16 (18.82%)	13 (9.49%)	50 (9.12%)	79 (10.26%)
46-50 years	16 (18.82%)	13 (9.49%)	32 (5.84%)	61 (7.92%)
51-55 years	20 (23.53%)	27 (19.71%)	18 (3.28%)	65 (8.44%)
56-60 years	7 (8.24%)	23 (16.79%)	36 (6.57%)	66 (8.57%)
>60 years	16 (18.82%)	12 (8.76%)	24 (4.38%)	52 (6.75%)
Total	85 (100.0%)	137 (100.0%)	548 (100.0%)	770 (100.0%)

The comparison of sternal dimensions between genders revealed statistically significant differences in most measurements. The mean length of the manubrium was significantly greater in males (51.21 ± 4.79 mm) compared to females (46.93 ± 4.77 mm, p = 0.000). Similarly, the mean length of the mesosternum was more extended in males (92.28 ± 9.78 mm) than in females (76.93 ± 7.51 mm; p = 0.000). The combined manubrium and mesosternum length was also significantly greater in males (143.50 ± 11.30 mm) compared to females (123.86 ± 9.52 mm; p = 0.000). The width of the first sternebra (S1W) was significantly larger in males (30.00 ± 24.30 mm) than in females (25.49 ± 4.71 mm; p = 0.028). The width of the third sternebra (S3W) was also significantly higher in males (28.80 ± 5.65 mm) than in females (24.73 ± 4.28 mm; p = 0.000). However, despite size differences, no significant difference was observed in the relative width index between genders (p = 0.689), indicating proportional similarity in sternal width (Table 5).

**Table 5:** Average comparison for key sternal measurements by gender * shows significant p-values; p<0.05 was considered significant An unpaired (independent samples) t-test was used to compare the mean sternal measurements between males and females.

Sternal variable	Gender	Mean ± SD (mm)	Student t-test value	p-value
Length of manubrium	Female	46.93 ± 4.77	-8.90	0.000*
	Male	51.21 ± 4.79		
Length of mesosternum	Female	76.93 ± 7.51	-16.67	0.000*
	Male	92.28 ± 9.78		
Combined manubrium + mesosternum length	Female	123.86 ± 9.52	-18.05	0.000*
	Male	143.50 ± 11.30		
Width of first sternebra (S1W)	Female	25.49 ± 4.71	-2.20	0.028*
	Male	30.00 ± 24.30		
Width of third sternebra (S3W)	Female	24.73 ± 4.28	-7.67	0.000*
	Male	28.80 ± 5.65		
Relative width index (S1/S3 × 100)	Female	104.4	-0.40	0.689
	Male	105.6		

The regression analysis showed significant positive associations between several sternal parameters and the dependent variable (likely age, height, or another biological variable, depending on your study context). The length of the manubrium (B = 0.040; p = 0.000), length of the mesosternum (B = 0.028; p = 0.000), and combined manubrium + mesosternum length (B = 0.311; p = 0.000) were all significantly predictive. Additionally, both the width of the first sternebra (S1W) (B = 0.002; p = 0.001) and the width of the third sternebra (S3W) (B = 0.039; p = 0.000) showed significant positive regression coefficients. However, the relative width index did not show a significant relationship (p = 0.689), suggesting that this parameter is not a meaningful predictor in this context (Table 6).

**Table 6:** Regression analysis of gender with sternal measurements * shows significant p-values; p<0.05 was considered significant.

Sternal parameter	Regression coefficient (B)	Constant	p-value
Length of manubrium	0.040	-0.422	0.000*
Length of mesosternum	0.028	-0.862	0.000*
Combined manubrium + mesosternum length	0.311	-1.624	0.000*
Width of first sternebra (S1W)	0.002	1.470	0.001*
Width of third sternebra (S3W)	0.039	0.505	0.000*
Relative width index	0.000	1.541	0.689

## Discussion

In the present study, the association between gender and the fusion of the manubrium with the sternal body across various age groups was evaluated. No statistically significant association was observed between age and fusion of the manubrium with the body (p > 0.05) in the age groups <15 years, 16-20 years, 21-25 years, 26-30 years, 31-35 years, 41-45 years, and 46-50 years. However, a statistically significant association (p < 0.05) was found in the age groups 36-40 years, 51-55 years, 56-60 years, and above 60 years, indicating that the fusion of the manubrium with the body is influenced by gender in these age groups. The mean age of onset of fusion was 44 years in males and 41.34 years in females, while the mean age for complete fusion was 47.70 years in males and 50.82 years in females. Although the findings suggest that fusion may begin between 16 and 20 years, no significant overall association was found between age and fusion status (p > 0.05). The highest frequency of complete fusion was observed in individuals over 60.

In contrast to the present findings, Kaneriya et al. [13] reported that no cases below 40 years exhibited complete or partial fusion of the manubrium and sternal body in their study. They observed that complete fusion occurred only in individuals older than 50, with some degree of fusion (partial or complete) present in all individuals above 55. Similarly, Gautam et al. [14] reported that fusion began around 40 years of age and was complete by 55 years of age. They concluded that fusion was age-dependent but not influenced by sex, contrasting with the present findings.

Jit et al. [15], in their study of populations from Punjab, Haryana, and Himachal Pradesh, observed that complete manubriosternal fusion did not occur before the age of 21 years. They also reported non-fusion in 11.4% of males above 66 years and 37.5% of females over 40 years - findings that partially align with the current study. Singh et al. [11], studying the Manipuri population, found that fusion began at 26 years in males and 31 years in females, with complete fusion typically occurring after 50 years in both sexes. They also noted that males tended to fuse earlier than females.

Wadhawan et al. [16], in their study of the Delhi population, reported the mean age of onset of manubriosternal fusion as 42.6 ± 4.33 years in males and 42.12 ± 3.27 years in females, with complete fusion occurring at 65.81 ± 10.68 years in males and 58.36 ± 5 years in females, indicating earlier fusion in females, which is consistent with the present findings. Das [17] reported that fusion began around 28 years and was completed by 60 years. Still, they emphasized that the process was highly variable and unreliable - an observation consistent with the present study. Bardale [18], in his textbook on forensic medicine and toxicology, also noted that complete fusion typically occurs by the age of 60.

The present study found a statistically significant association between age and the fusion of the second to the first sternal segments (p < 0.05), suggesting that fusion of these segments is age-dependent. Fusion was observed to begin before puberty and was generally completed by the age of 35 years, independent of sex. Similar findings were reported by Kaneriya et al. [13] in their study of the Surat district population, where fusion of the first and second sternebrae began around puberty and was completed by 25 years of age, independent of sex. Jit et al. [15], studying the Ahmedabad population, also found that fusion was complete by 21 years and was not sex-dependent, supporting the results of the current study.

When compared with previous studies, Wadhawan et al. [16] reported mean ages close to those observed in the present study, while Kaneriya et al. [13] and Gautam et al. [14] reported slightly earlier ages, around 40 years. The mean age of complete manubriosternal fusion in the present study was 47.70 years in males and 50.82 years in females.

The mean age of onset for the fusion of the xiphoid process with the mesosternum in the present study was 43.70 years in males and 49.67 years in females. However, earlier ages for the onset of fusion were reported by Wadhawan et al. [16], Kaneriya et al. [13], and Dkhar [19], highlighting population-level variability in fusion timing.

Limitations of the study

Despite the valuable insights provided by this study, certain limitations should be acknowledged. First, the sample was drawn exclusively from a single tertiary care institution in Central India, which may limit the generalizability of the findings to other regional or ethnic populations. Genetic, nutritional, and environmental factors can influence skeletal development and fusion patterns; thus, population-specific variations may exist that are not captured in this dataset.

Second, while efforts were made to exclude sternum with visible deformities, fractures, or pathological lesions, the internal bone architecture and microscopic ossification processes were not assessed. Radiographic or histological evaluation could have provided additional confirmation regarding the fusion stages, especially in borderline or ambiguous cases.

Third, age estimation relied on official records and familial accounts provided by law enforcement and relatives, which may not always be precise, particularly in cases involving unclaimed or unidentified bodies. Inaccuracies in reported age could potentially influence the statistical associations between age and fusion status.

Fourth, the study employed only gross macroscopic examination to assess fusion status, categorizing it as complete, partial, or absent. This method, while practical, is inherently subjective and may be prone to inter-observer variability. More objective techniques, such as CT imaging or digital morphometric analysis, could improve accuracy and reproducibility.

To validate and extend the current findings, future studies incorporating a multicentric, radiological, and possibly histological approach, along with larger and more diverse samples, are recommended.

## Conclusions

The present study's findings highlight that the sternum exhibits progressive, age-related morphological changes, particularly in the fusion patterns of its components. A statistically significant association was observed between age and the fusion of the second to first sternebra, with complete fusion typically occurring by 35 years of age, making it a valuable marker for age estimation in younger individuals. The observed mean ages for the onset and completion of sternal fusion in this Central Indian population differed from those reported in other regional studies, emphasizing the need for population-specific anthropometric standards. While sternal fusion is a valuable indicator for age estimation, its variability - especially in older age groups - necessitates cautious interpretation and should be corroborated with data from other skeletal structures. Given its resistance to decomposition and frequent recovery in fragmented remains, the sternum remains a crucial element in forensic examinations. Its forensic utility is significantly enhanced when incorporated into a multi-factorial assessment that includes the pelvis, skull, and long bones. This study contributes a preliminary dataset on age-related sternal fusion trends for the Central Indian population, thereby addressing a critical gap in the existing forensic literature.
